# Distal locking mechanism influences surgical and radiological outcomes in proximal femoral nailing using distal wedge versus distal screw designs

**DOI:** 10.1038/s41598-025-08079-y

**Published:** 2025-07-10

**Authors:** Aytek Hüseyin Çeliksöz, Büşra Tokmak, Ali Okan Tarlacık, Servet Igrek

**Affiliations:** 1 Orthopedics and Traumatology, Eskişehir City Hospital, 26000 Eskişehir, Turkey; 2 Orthopedics and Traumatology, Igdir State Hospital, Iğdır, Turkey; 3 Orthopedics and Traumatology, Kartal Education and Research Hospital, 34000 Istanbul, Turkey; 4 Orthopedics and Traumatology, Koç University Hospital, 34000 Istanbul, Turkey

**Keywords:** Proximal femoral nail, Intertrochanteric fracture, Distal locking, Wedge locking, Fracture union, Medical research, Outcomes research

## Abstract

This study aims to evaluate the radiological outcomes of two proximal femoral nails that share similar proximal geometry and sizes, but differ in their distal locking mechanisms. This retrospective study included 244 patients with AO 31-A1/A3 intertrochanteric fractures treated with either Wedge-wing proximal femoral nail (Ww-PFN) (*n* = 158) or Distally Wedge proximal femoral nail (Dw-PFN) (*n* = 86). Radiological parameters such as fracture reduction quality, tip-apex distance (TAD), neck-shaft angle (NSA) and time to fracture healing were compared. The Dw-PFN group demonstrated significantly shorter fracture healing time (12.45 ± 9.7 vs. 15.0 ± 3.4 months, *p* < 0.001) and better fracture reduction quality (*p* < 0.001) compared to the Ww-PFN group. NSA decreased in both groups postoperatively, with a greater mean decrease observed in the Dw-PFN group; this difference was not statistically significant (*p* = 0.175). However, complication rates were not different. (*p* = 0.342). The expandable talon mechanism of the Dw-PFN was associated with significantly faster fracture healing and shorter surgical duration compared to the Ww-PFN system. The mean surgical time was 34.1 ± 6.5 min for the Dw-PFN group and 50.6 ± 10.4 min for the Ww-PFN group (*p* = 0.001). These findings suggest that the talon-type distal fixation may be a favorable alternative in clinical practice. Notably, despite the lower rate of good fracture reduction in the Dw-PFN group (28.5% vs. 71.3%), complication and failure rates were comparable, further supporting the safety and clinical feasibility of this locking mechanism.

## Introduction

With an aging global population, intertrochanteric femur fractures represent a growing healthcare burden, with incidence projected to reach 10 million cases by 2050^1^. Surgical intervention is typically necessary to restore function and reduce associated complications^2^.

Biomechanical studies have shown that intramedullary implants may offer improved resistance to mechanical failure due to shorter lever arms and centralized load transmission, particularly under cyclic loading conditions in pertrochanteric fractures^3^. Several intramedullary devices  (e.g.  the Gamma Nail, DLT, Talon Distal Fix, InterTan, and Double Lag Screw - have been introduced, each with distinct locking mechanisms. However, no single implant has consistently proven to be superior in clinical and radiological outcomes^3–6^.

The aim of this study is to compare the radiological outcomes of two proximal femoral nails with similar proximal geometry and dimensions but different distal locking mechanisms. Specifically, this study will investigate how two distinct distal fixation techniques - Dw-PFN and Ww-PFN - affect fracture stability, time to union, and complication rates in intertrochanteric fractures. It is hypothesized that the Dw-PFN, despite having similar fracture patterns and mechanical failure rates to the Ww-PFN, will result in a shorter time to union, potentially due to differences in biomechanical stability and load distribution.

## Materials and methods

This study is retrospective in nature, and the requirement for informed consent was waived by the Non-Interventional Clinical Research Ethics Committee of Eskişehir City Hospital (T.C. Eskişehir Şehir Hastanesi) in accordance with national regulations. All procedures were carried out in accordance with relevant institutional guidelines, and in compliance with the ethical principles outlined in the Declaration of Helsinki. A retrospective analysis was performed on 244 patients who were diagnosed with intertrochanteric femur fractures and admitted between 2018 and 2021 at two tertiary referral hospitals.

Inclusion criteria were defined as patients aged 65 years or older, diagnosed with intertrochanteric fractures classified as AO 31A1 to 31A3, who underwent osteosynthesis with either the distal screw locking PFN or the distal wedge locking PFN.

Patients were excluded if they were younger than 65 years, had ipsilateral femoral shaft or distal femoral fractures, presented with pathological fractures, had previously undergone surgical intervention on the ipsilateral limb, or had comorbities leading to prolonged immobilization. All intertrochanteric fractures were treated exclusively with Ww-PFN at one hospital and Dw-PFN at the other.


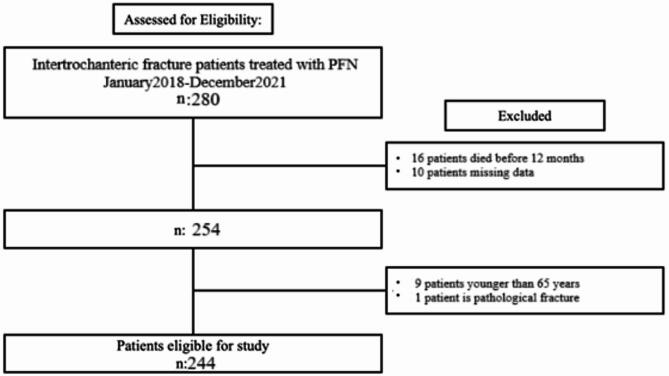


### PFN types and features

#### Dw-PFN features

The Dw-PFN is available in lengths ranging from 220 mm to 420 mm, with 20 mm increments. The distal portion of the nail has a diameter of 11 mm, while the proximal portion measures 15.5 mm in diameter. The nail features a 4° lateral bend angle.

**Fig. 1:**
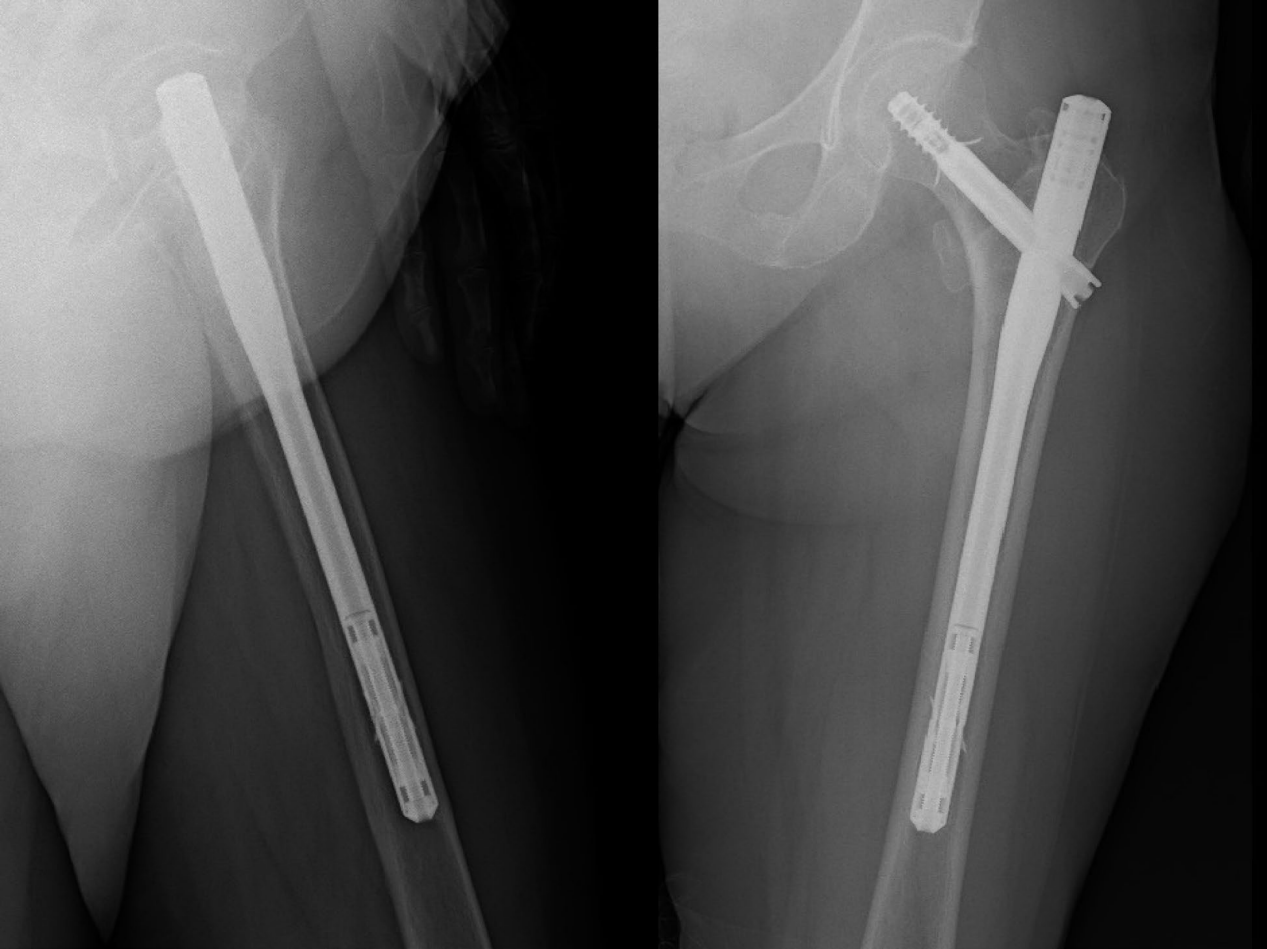
Sample case treated with Dw-PFN.

For proximal fixation, a single lag screw with four deployable/retractable claws (maximum deployable diameter: 28 mm) can be inserted at 120°, 125°, or 130°. The lag screw is available in lengths ranging from 70 mm to 120 mm, with 5 mm increments. It has a thread diameter of 11 mm and a root diameter of 8.2 mm. Distal locking does not require an additional incision, as the nail is equipped with six deployable/retractable claws which expand to a total diameter of 38 mm at the distal end. The distal locking mechanism and nail design can be observed in the postoperative radiographs presented in Fig. 1.

#### Ww-PFN (wedge wing-PFN)

The wedge-wing PFN (Ww-PFN) is available in lengths ranging from 220 mm to 420 mm, with 20 mm increments. The distal portion of the nail has a diameter of 11 mm, while the proximal portion measures 16.5 mm in diameter. The implant features a lateral bend angle of 6°.

For proximal fixation, a single lag screw with four deployable/retractable claws, capable of expanding to a maximum diameter of 28 mm, can be inserted at angles of 120°, 125°, and 130°. The lag screw is available in lengths ranging from 70 mm to 120 mm, with 5 mm increments, and has a thread diameter of 11 mm and a root diameter of 8.2 mm.

**Fig. 2:**
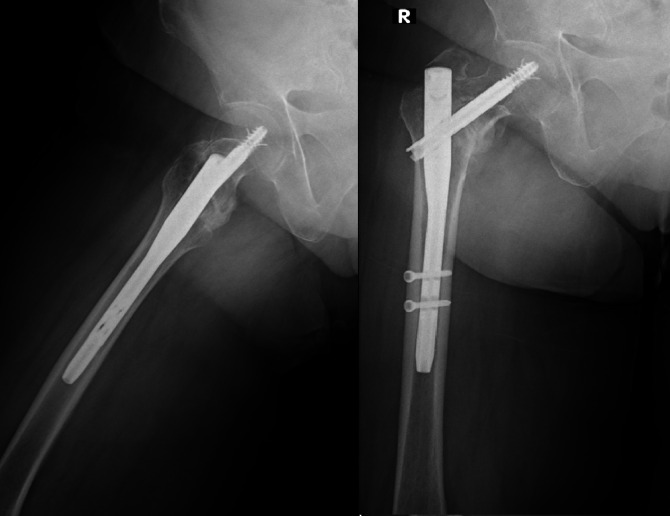
Sample case treated with Ww-PFN.

Unlike the Dw-PFN, the Ww-PFN incorporates two screws with 5 mm thread diameters for distal locking, providing an alternative stabilization mechanism. The distal locking mechanism and nail design can be observed in the postoperative radiographs presented in Fig. 2.

### Surgery technique

Patients were positioned in either the supine or lateral decubitus position depending on the surgical setup or table availability. After site preparation and draping, reduction was achieved via manual traction or traction table. A proximal entry was created at the center of greater trochanter, and the intramedullary nail was inserted after determining the appropriate length. Lag screws were placed in a center-center or inferior-center position under fluoroscopic guidance^8^. In the Ww-PFN group, the lag screw’s claws were deployed, and distal locking was achieved using cortical screws. In the Dw-PFN group, the distal talons and lag screw talons were deployed sequentially. Final implant positioning was confirmed fluoroscopically.

Postoperatively, all patients were mobilized with a walker, bearing weight as tolerated, and followed up at 1, 3, 6, and 12 months to assess clinical and radiographic outcomes.

### Data evaluation

The following demographic and surgical variables were analyzed: age, sex, duration of surgery (defined as the time from skin incision to skin closure), total hospital stay, time from admission to surgery, and bone mineral density (BMD) measurements assessed via DEXA. Additionally, patient positioning during surgery and the need for revision surgery were recorded.

Radiological parameters were evaluated as defined below. All measurements were independently performed by two authors who participated in the surgical procedures. The assessment included:

Fracture classification, performed according to the AO/OTA system (31-A1 to 31-A3)^9^.

Quality of fracture reduction, evaluated using Baumgaertner’s criteria, categorizing reductions as “good,” “acceptable,” or “poor.” A good or acceptable reduction was defined as normal or slight valgus alignment on anteroposterior X-rays, less than 20° angulation on lateral X-rays, and fragment displacement of less than 4 mm^9,10^.

Tip-apex distance (TAD,mm), was measured as the sum of the distances from the tip of the lag screw to the apex of the femoral head on both anteroposterior and lateral radiographs^11,12^.

Early and late postoperative neck-shaft angle measurements, which are defined as the angle between the anatomical axis of the femoral neck and the anatomical axis of the femoral diaphysis. Neck-shaft angle and TAD measurements were performed using radiographs obtained on the first postoperative day, while reduction quality was also assessed at the same time.

Fracture healing was evaluated radiographically and defined as the presence of bridging callus in at least three out of four cortices on anteroposterior and lateral radiographs^14^.

Occurrence of mechanical complications, including cut-out and implant failure: The two usual modes of failure in PFN treatment involve the collapse of the neck-shaft angle into varus, leading to screw extrusion, also known as cut-out, or the medial migration of the lag screw, referred to as cut-through^11,14,15^.

### Statistical analysis

All statistical analyses were performed using SPSS software, version 25.0 (IBM Corp., Armonk, NY, USA). Descriptive statistics were used to summarize the data, with continuous variables presented as mean ± standard deviation (SD) and median values, while categorical variables were expressed as frequencies and percentages. The Student’s t-test was used for normally distributed data, while the Mann-Whitney U test was applied for non-normally distributed variables. The Pearson chi-square test was used for categorical data comparisons. A p-value less than 0.01 was considered statistically significant.

## Results

The clinical outcomes are presented in Table 1. In this study, a total of 244 patients were included, with 158 in the Ww-PFN group and 86 in the Dw-PFN group. The median age was 79 years in the Ww-PFN group (range: 65–103) and 78 years in the Dw-PFN group (range: 65–92), with no statistically significant difference between the groups (*p* = 0.213).

Regarding gender distribution, 56.3% (*n* = 89) of patients in the Ww-PFN group and 51.7% (*n* = 45) in the Dw-PFN group were female. The gender distribution between the two groups did not show a statistically significant difference (*p* = 0.488) (Table 1).

The mean duration of surgery was significantly shorter in the Dw-PFN group (34.12 ± 6.54 min) compared to the Ww-PFN group (50.64 ± 10.42 min) (*p* < 0.001) (Table 1).

The time from hospital admittance to surgery was significantly different between the two groups (*p* < 0.001). In the Dw-PFN group, the majority of patients (58.6%) underwent surgery within the first 24 h, whereas only 0.6% of patients in the Ww-PFN group underwent surgery within the same time frame. Most patients in the Ww-PFN group (60.1%) underwent surgery between 24 and 48 h, while this percentage was 26.4% in the Dw-PFN group. Furthermore, 39.2% of patients in the Ww-PFN group had a surgery after 48 h, compared to only 14.9% in the Dw-PFN group. This statistically significant difference means that patients treated with Dw-PFN were more likely to receive earlier surgical intervention compared to those treated with Ww-PFN.

The mean hospital stay was significantly shorter in the Ww-PFN group (3.86 ± 1.8 days) compared to the Dw-PFN group (6.89 ± 3.98 days) (*p* < 0.001) (Table 1).

In the Ww-PFN group, 31% of patients had normal bone density, 22.2% were osteopenic, and 46.8% were osteoporotic. In the Dw-PFN group, 19.5% of patients had normal bone density, 28.7% were osteopenic, and 51.7% were osteoporotic. Bone mineral density (BMD) classification did not show a statistically significant difference between the Ww-PFN and Dw-PFN groups (*p* = 0.135) (Table 2).

The Ww-PFN system was more commonly performed using a traction table or lateral decubitus positioning, while the Dw-PFN system was predominantly performed in the supine position (*p* < 0.001) (Table 2).

Revision surgery was required in 12.7% of patients in the Ww-PFN group, while the rate was lower in the Dw-PFN group at 6.9%. However, this difference was not statistically significant (*p* = 0.161). Overall, 89.4% of patients did not require revision surgery (Table 2).

**Table 1:** Comparison of clinical outcomes between Ww-PFN and Dw-PFN Groups.

	Ww-PFN (*n* = 158)	Dw-PFN (*n* = 86)	*p* value
Age			
Mean ± SD	78.81 ± 9.47	77.30 ± 8.26	0.213
Median (Min−Max)	79 (65–103)	78 (65–92)	
Gender			
Female	89 (56.3%)	45 (51.7%)	0.488
Male	69 (43.7%)	42 (48.3%)	
Surgery duration			
Mean ± SD	50.64 ± 10.42	34.12 ± 6.54	0.001**
Median (Min−Max)	50 (30–90)	34 (19–60)	
Length of hospital stay			
Mean ± SD	3.86 ± 1.8	6.89 ± 3.98	0.001**
Median (Min−Max)	4 (2–20)	5 (3–20)	

Radiologically, results present in the Table 2. The assessment included: According to the AO/OTA classification, AO 31.A2 fractures were the most common type, observed in 74.1% of patients in Ww-PFN group and 62.1% of patients in the Dw-PFN group, with a total incidence of 69.8%. The distribution of fracture types between the two groups didn’t show a statistically significant difference (*p* = 0.085).

Based on Baumgaertner’s criteria, good reduction was achieved in 28.5% of patients in the Ww-PFN group and 71.3% of patients in the Dw-PFN group; acceptable reduction was observed in 58.9% of Ww-PFN patients and 17.2% of Dw-PFN patients and poor reduction was noted in 12.7% of Ww-PFN patients and 11.5% of Dw-PFN patients, with an overall incidence of 12.2%. A statistically significant difference was found between the two groups (*p* < 0.001), with the Dw-PFN group achieving a higher rate of good reduction compared to the Ww-PFN group.

The mean TAD was 23.1 ± 5.18 mm in the Ww-PFN group, while it was 28.78 ± 8.37 mm in the Dw-PFN group. The Ww-PFN group remained within the target range, whereas the Dw-PFN group exceeded the threshold on average, and this difference was statistically significant (*p* < 0.001).

The initial neck-shaft angle (NSA) was 129.46° ± 3.37° in the Ww-PFN group and 128.49° ± 4.77° in the Dw-PFN group. At the final follow-up, the mean NSA decreased to 127.59° ± 4.14° in the Ww-PFN group and 126.24° ± 5.7° in the Dw-PFN group. In the late postoperative measurements, based on early measurements, a decrease of 1.87° was observed in the Ww-PFN group, while a decrease of 2.25° was noted in the Dw-PFN group in the later assessments (*p* = 0.175).

Fracture healing time was significantly shorter in the Dw-PFN group (12.45 ± 9.72 months) compared to the Ww-PFN group (15.06 ± 3.41 months) (*p* < 0.001).

Complication rates were comparable between the two groups (*p* = 0.342), with complications occurring in 13.3% of Ww-PFN patients and 9.2% of Dw-PFN patients. Although complications occurred earlier in the Dw-PFN group, the difference in complication rates and timing between the two groups was not statistically significant. In the Ww-PFN group, one patient experienced distal wedge breakage, and in the Dw-PFN group, two patients had distal screw fractures. These events did not compromise fracture stability or require revision surgery, and therefore were classified as complications without reoperation.

**Table 2:** Comparison of radiological outcomes between Ww-PFN and Dw-PFN groups.

Parametreler	Ww-PFN (*n* = 158)	Dw-PFN (*n* = 86)	*p* value
Bone density			
Normal	49 (31.0%)	17 (19.5%)	0.135
Osteopenic	35 (22.2%)	25 (28.7%)	
Osteoporotic	74 (46.8%)	45 (51.7%)	
Reduction quality			
Good	45 (28.5%)	62 (71.3%)	0.001**
Acceptable	93 (58.9%)	15 (17.2%)	
Poor	20 (12.7%)	10 (11.5%)	
Tip apex distance (mm)			
Mean ± SD	23.1 ± 5.18	28.78 ± 8.37	0.001**
Median (Min-Max)	25 (10–35)	26 (10–51)	
Patient position			
Lateral decubitus	46 (29.1%)	22 (25.3%)	0.001**
Supine position	36 (22.8%)	65 (74.7%)	
Traction table	76 (48.1%)	0 (0.0%)	
Initial neck shaft angle			
Mean ± SD	129.46 ± 3.37	128.49 ± 4.77	0.044*
Median (Min-Max)	130 (120–135)	129 (116–140)	
Follow-up neck shaft angle			
Mean ± SD	127.59 ± 4.14	126.24 ± 5.7	0.175
Median (Min-Max)	130 (115–135)	128 (108–140)	
Fracture type			
AO 31.1	28 (17.7%)	26 (29.9%)	0.085
AO 31.2	117 (74.1%)	54 (62.1%)	
AO 31.3	13 (8.2%)	7 (8.0%)	
Complications			
None	137 (86.7%)	79 (90.8%)	0.342
Present	21 (13.3%)	8 (9.2%)	
Revision surgery			
No	138 (87.3%)	81 (93.1%)	0.161
Yes	20 (12.7%)	6 (6.9%)	
Fracture healing time (Mo)			
n	150	80	0.001**
Mean ± SD	15.06 ± 3.41	12.45 ± 9.72	
Median (Min-Max)	14 (9–28)	11.5 (4–60)	

## Discussion

The most significant finding of this study was that the Dw-PFN design significantly accelerated faster fracture healing time compared to the Ww-PFN. Additionally, the Dw-PFN design outperformed the Ww-PFN in terms of reduced surgical duration.

The faster fracture healing observed in our study, which focused on the distal locking method, can be attributed not only to the high stability provided by the claw-retractable mechanism^17^ but also to micro-movements reported in biomechanical studies^18^. Previous reports comparing three different PFN designs (Talon, PFNA, Intertan) indicated that variations in lag screw properties did not result in significant differences in radiological healing times^19^. In this study, the shorter fracture healing time observed in the Dw-PFN group appears to be consistent with this reported stability in the literature.

The quality of fracture reduction is a critical factor in the success of fracture management and plays a key role in the overall effectiveness of PFN fixation^9,19^. In this study, adequate fracture reduction was achieved in both groups; however, good reduction was more frequently observed in the Dw-PFN group.^20,21^.

The necessity of distal locking is debated in the literature. Some studies suggest that distal locking should not be used in AO 31-A1 or A2 fractures^22–24^. While others argue that distal locking is more beneficial in unstable fractures and prevents complications^26^. Different complications associated with the distal locking system have been reported, ranging from femoral bone fractures and vascular injuries to prolonged surgical time. The Dw-PFN offers several advantages, including its minimally invasive technique, ease of distal locking, reduced operation time, and absence of distal screw complications^27^.

One of the primary strengths of this study is the comparison of two groups with matched demographic characteristics and bone quality, allowing for the focused evaluation of a single variable - distal locking - within an otherwise similar nail design. These findings provide valuable insights that may guide implant selection in clinical practice, potentially enhancing patient outcomes in the management of intertrochanteric fractures.

This study is subject to several limitations. First, measurements and surgeries were performed by two different surgeons, which may have introduced variability in both technique and interpretation. Second, certain key parameters, such as reduction quality and tip-apex distance, may have varied and potentially influenced the study outcomes. Finally, the retrospective design inherently carries the risk of selection bias and limits the ability to establish causal relationships.

Additionally, the significant difference in postoperative reduction quality between groups may have influenced the rate of fracture healing. Although reduction quality was not the primary focus of this study, its potential impact on outcomes should be considered when interpreting the results.

Surgical timing was influenced by institutional workflow and patient-related factors, which may have affected the length of hospital stay.

These findings provide valuable insights into the impact of distal locking mechanisms on fracture healing and may aid in optimizing implant selection for intertrochanteric fractures. However, further studies with larger sample sizes and longer follow-up periods are required to confirm these results and determine whether these advantages translate into improved better long-term functional outcomes for patients.

## Data Availability

The datasets analysed during the current study are not publicly available due to the inclusion of identifiable patient information, such as hospital admission and discharge dates, and the involvement of specific healthcare institutions. However, de-identified data may be shared by the principal investigator, Aytek Hüseyin Çeliksöz, upon reasonable request and with institutional approval. If you want to examine the rawdata personally, we can apply for the institutional approval via our principal investigator. He can be reached through his mail, aceliksoz@kuh.ku.edu.tr.

## References

[1] Wu, A. M. et al. Global, regional, and National burden of bone fractures in 204 countries and territories, 1990–2019: a systematic analysis from the global burden of disease study 2019. *Lancet Healthy Longev.* **2** (9), e580–e592 (2021). 34723233 10.1016/S2666-7568(21)00172-0 PMC8547262

[2] Xu, Z., Zhang, M., Yin, J., Ren, L. & Zeng, Y. Redisplacement after reduction with intramedullary nails in surgery of intertrochanteric fracture: cause analysis and preventive measures. *Arch. Orthop. Trauma Surg.* **135**, 751–758. 10.1007/s00402-015-2205-y (2015). 10.1007/s00402-015-2205-y 25808352

[3] Schader, J. F. et al. Biomechanical comparison of intramedullary versus extramedullary implants for fixation of simple Pertrochanteric fractures. *J. Orthop. Trauma.* **37** (5), 243–248 (2023). 36728969 10.1097/BOT.0000000000002552

[4] Yapici, F. et al. Clinical and radiological outcomes of patients treated with the Talon distalfix proximal femoral nail for intertrochanteric femur fractures. *Injury* **51** (4), 1045–1050 (2020). 32151425 10.1016/j.injury.2020.03.006

[5] Kürüm, H. et al. Intertrochanteric femoral fractures: A comparative analysis of clinical and radiographic outcomes between talon intramedullary nail and intertan nail. *Cureus* **15**, 12 (2024). 10.7759/cureus.50877 PMC10801105 38259364

[6] Arıcan, G. et al. Talon proksimal femoral Çivileme (Pfn) proksimal femoral Çivi-Antirotasyon (Pfna) Kadar Başarılı mı?? *Acta Med. Alanya*. **3** (3), 261–266 (2019).

[7] Duramaz, A. & İlter, M. H. The impact of proximal femoral nail type on clinical and radiological outcomes in the treatment of intertrochanteric femur fractures: a comparative study. *Eur. J. Orthop. Surg. Traumatol.* **29**, 1441–1449. 10.1007/s00590-019-02454-y (2019). 10.1007/s00590-019-02454-y 31147767

[8] Cleveland, M., Bosworth, D. M., Thompson, F. R., Wilson, H. J. & Ishizuka, T. A ten-year analysis of intertrochanteric fractures of the femur. *JBJS* **41** (8), 1399–1408 (1959). 13849408

[9] Meinberg, E. G., Agel, J., Roberts, C. S., Karam, M. D. & Kellam, J. F. Fracture and dislocation classification compendium—2018. *J. Orthop. Trauma.* **32**, S1–S10 (2018). 29256945 10.1097/BOT.0000000000001063

[10] Park, Y. C., Yoon, S. P. & Yang, K. H. The effects of extramedullary reduction in unstable intertrochanteric fracture: a Biomechanical study using cadaver bone. *J. Korean Fract. Soc.* **31** (3), 79–86 (2018).

[11] Baumgaertner, M. R., Curtin, S. L. & Lindskog, D. M. Intramedullary versus extramedullary fixation for the treatment of intertrochanteric hip fractures. *Clin. Orthop. Relat. Res.* **348**, 87–94 (1998). 9553538

[12] Baumgaertner, M. R., Curtin, S. L., Lindskog, D. M. & Keggi, J. M. The value of the tip-apex distance in predicting failure of fixation of peritrochanteric fractures of the hip. *JBJS* **77** (7), 1058–1064 (1995). 10.2106/00004623-199507000-00012 7608228

[13] Geller, J. A., Saifi, C., Morrison, T. A. & Macaulay, W. Tip-apex distance of intramedullary devices as a predictor of cut-out failure in the treatment of peritrochanteric elderly hip fractures. *Int. Orthop.* **34**, 719–722. 10.1007/s00264-009-0837-7 (2010). 10.1007/s00264-009-0837-7 PMC2903170 19618186

[14] Shin, W. C. et al. Radiographic outcomes of osteosynthesis using proximal femoral nail antirotation (PFNA) system in intertrochanteric femoral fracture: has PFNA II solved all the problems?? *Hip Pelvis*. **29** (2), 104–112 (2017). 28611961 10.5371/hp.2017.29.2.104 PMC5465391

[15] Flint, J. H., Sanchez-Navarro, C. F., Buckwalter, J. A. & Marsh, J. L. Intrapelvic migration of a gamma nail lag screw: review of the possible mechanisms. *Orthopedics* **33**, 266–270. 10.3928/01477447-20100225-19 (2010). 10.3928/01477447-20100225-19 20415308

[16] Liu, W., Zhou, D., Liu, F., Weaver, M. J. & Vrahas, M. S. Mechanical complications of intertrochanteric hip fractures treated with trochanteric femoral nails. *J. Trauma. Acute Care Surg.* **75** (2), 304–310 (2013). 23887564 10.1097/TA.0b013e31829a2c43

[17] Aycan, M. Comparison of biomechanical properties of implant systems used in treatment of proximal femur fractures, *J. Fac. Eng. Archit. Gazi Univ.* **34**, 2 (2019).

[18] Bramlet, D. G. & Wheeler, D. Biomechanical evaluation of a new type of hip compression screw with Retractable talons. *J. Orthop. Trauma.* **17** (9), 618–624 (2003). 14574189 10.1097/00005131-200310000-00004

[19] Zehir, S., Şahin, E. & Zehir, R. Comparison of clinical outcomes with three different intramedullary nailing devices in the treatment of unstable trochanteric fractures. *Turk. J. Trauma. Emerg. Surg.*. **21** (6), 469–476 (2015). 10.5505/tjtes.2015.28227 27054638

[20] Ito, J. et al. Prevention of excessive postoperative sliding of the short femoral nail in femoral trochanteric fractures. *Arch. Orthop. Trauma. Surg.* **135** (5), 651–657. 10.1007/s00402-015-2200-3 (2015). 10.1007/s00402-015-2200-3 25801809

[21] Cheng, Y. C. Construction of a risk prediction model to identify potential risk factors for the development of delirium after total hip arthroplasty. *Med. (Baltim).* **103** (52), e41054 (2024). 10.1097/MD.0000000000041054 PMC11688097 39969388

[22] Birkner, D. et al. The vulnerability of hip fracture patients with cognitive impairment: an analysis of health conditions, hospital care, and outcomes. *BMC Geriatr.* **25** (1), 99 (2025). 39953428 10.1186/s12877-025-05744-9 PMC11829398

[23] Ozkan, K., Unay, K., Demircay, C., Cakir, M. & Eceviz, E. Distal unlocked proximal femoral intramedullary nailing for intertrochanteric femur fractures. *Int. Orthop.* **33**, 1397–1400. 10.1007/s00264-008-0673-1 (2009). 10.1007/s00264-008-0673-1 PMC2899111 18956183

[24] Skála-Rosenbaum, J., Bartoníček, J. & Bartoška, R. Is distal locking with IMHN necessary in every Pertrochanteric fracture? *Int. Orthop.* **34** (7), 1041–1047. 10.1007/s00264-009-0874-2 (2010). 10.1007/s00264-009-0874-2 PMC2989034 19882156

[25] Li, X. et al. Distal locked and unlocked nailing for perthrochanteric fractures—a prospective comparative randomized study. *Int. Orthop.* **39** (8), 1645–1652. 10.1007/s00264-015-2771-1 (2015). 10.1007/s00264-015-2771-1 25913263

[26] Tisherman, R. T., Hankins, M. L., Moloney, G. B. & Tarkin, I. S. Distal locking of short cephalomedullary nails decreases varus collapse in unstable intertrochanteric fractures–a Biomechanical analysis. *Injury* **52** (3), 414–418 (2021). 33593524 10.1016/j.injury.2021.02.007

[27] Hegde, A., Khanna, V., Mane, P. & Shetty, C. A comparative analysis of distal locked and unlocked long proximal femoral nail antirotation (PFNA-II) in the fixation of stable intertrochanteric fractures. *Chin. J. Traumatol. -Engl Ed.* **26** (2), 111 (2023). 10.1016/j.cjtee.2022.12.007 PMC10071317 36635155

